# Significance and challenges in dissecting cancer-bacteriome interactions

**DOI:** 10.1038/s44276-026-00229-7

**Published:** 2026-05-04

**Authors:** Ibraheem Alshareedah, James D. Brunner, Patrick S. G. Chain, Anand Kumar

**Affiliations:** 1 Microbial and Biome Sciences group, Bioscience Division, Los Alamos National Laboratory, Los Alamos, NM USA; 2 Genomics and Bioanalytics group, Bioscience Division, Los Alamos National Laboratory, Los Alamos, NM USA

## Abstract

Cancer is the leading cause of death around the world. While some types of cancer have become manageable due to advancements in medicine, most cancers still lack available cures and treatments. Recent studies have shown that changes in the human microbiome, especially in the bacteriome, are associated with some cancers. Certain bacterial strains have been reported to promote the initiation and progression of cancer in humans. Other studies have used sequencing to observe changes in the bacteriome of healthy and cancer patients. However, studies that investigate the interactions between cancer cells and the complex bacteriome as a whole remain scarce. This is due to the absence of experimental methods to study the interactions between cancer cells and complex bacterial populations, which has delayed the progress in identifying cancer-causing and cancer-inhibiting bacteria, and in understanding the bacterial interactions and their influence on host cells. Here, we review approaches to studying cancer cell interactions with complex bacteriomes and suggest possible routes to overcome this problem, highlighting the need for interdisciplinary studies that may help advance this field. We speculate that a good understanding of cancer-bacteriome interactions may open the door to new lines of holistic bacteriotherapy for cancer that is otherwise unavailable.

## Introduction

The study of the human microbiome has attracted significant research interest in recent years [1–7]. This is due to accumulating evidence suggesting that the microbiome plays an important role in human health [8–10], which has led to many attempts to harness the microbiome for treating certain medical conditions, especially in the broader field of gastroenterology [11, 12]. Recently, it has been shown in multiple seminal studies that the microbiome is intimately connected with cancer [13–20]. Cancer patients have altered microbial populations compared to cancer-free patients [14, 21–23]. This suggests that there is a mode of interaction between cancer and the microbiome. Although we do not know whether all microbiome changes associated with cancer are causes for the latter [24], it is clear that cancer cells and microbial cells interact [25, 26]. Given the fact that many organs in the human body have distinct microbiome populations [3, 27, 28], understanding the interactions between specific organ cancer cells and the microbiome may lead to advances in the early detection and management of cancer. This opens a new frontier in the fight against cancer, one of the deadliest diseases in human history.

The human microbiome consists of three components: the virome (viruses), the bacteriome (bacteria), and the mycobiome (fungi). Of the three components, the bacteriome is the largest, accounting for more than 90% of all microbes hosted by the human body and ~50% of the human body cell content [29, 30]. However, even with many advancements in bacteriome studies, a significant portion of bacteria remain unclassified [31, 32], let alone the functions they perform. In the context of cancer, the bacteriome is of special interest because studies have shown that some bacterial taxa have direct effects on cancer initiation and progression [33–37] (Table 1). For example, extensive evidence supports that *Helicobacter pylori* has a direct causal link with gastric cancer, which has made this the only bacterial species that has been listed as a carcinogen by the World Health Organization [38–40]. *F. nucleatum* is another bacterial species that is found in unusually high abundance in microbial communities in colorectal and lung cancers [41–44]. Furthermore, certain strains of *E. coli* have been shown to produce genotoxins that induce DNA double-strand breaks that are associated with various types of cancers [45–49].

**Table 1:** List of bacteria that are associated with cancer types.

Bacterial species	Cancer type	References
*H. pylori*	Gastric Cancer, liver cancer, MALT lymphoma	[39, 167, 168]
*F. nucleatum*	Colorectal, Cervical, Lung, Pancreas	[41, 169]
*P. gingivalis*	Colorectal, Pancreas	[170, 171]
*Enterococcus faecalis*	Liver	[172]
*E. coli*	Lung, Colorectal	[173, 174]
*Streptococcus*	Prostate	[175]
*Staphylococcus epidermidis*	Lung, Breast	[176, 177]
*Enterobacteriaceae*	Breast	[177]
*S. yanoikuyae*	Breast	[178]
*Salmonella typhi*	Gallbladder	[179]
*Enterotoxigenic B. fragilis*	Colorectal	[180]
*Campylobacter jejuni*	Colorectal	[181]
*Peptostreptococcus anaerobius*	Colorectal	[182]
*Morganella morganii*	Colorectal	[183]
*Chlamydia pneumoniae*	Lung	[184]
*Salmonella* Enteritidis	Colon	[185]

Conversely, certain bacterial species have been associated with cancer-suppressive effects [50–53]. For example, *Bifidobacterium* has been shown to suppress tumor growth by stimulating a tumor-suppressive immune response [54]. Additionally, Salmonella has been shown to localize to multiple tumor types and induce apoptosis [55, 56]. *Listeria monocytogenes* also has cancer-suppressive properties as it activates the adaptive immune system against tumors and changes the tumor microenvironment [57–59]. This has spurred an exciting field of research that delves into engineering strategies to develop cancer bacteriotherapies [36, 50, 60, 61]. With that being said, the study of the interactions between bacterial populations and cancer cells remains in its infancy due to the complexity of the human bacteriome and the lack of suitable techniques to dissect these interactions at the cellular level.

The link between the bacteriome and cancer is of immense interest to microbiologists and medical scientists [62]. One of the remarkable features of the bacteriome is that it exists in almost every organ and/or tissue that can develop tumors [63]. Furthermore, bacteriome populations change depending on the location within the human body and are dynamic [27, 64], which offers a route to developing tissue-specific treatments that can be tailored to the patient’s bacteriome. This dichotomy between the general presence of the bacteriome and the specificity of the bacteriome population to different tissues offers a promise of personalized, yet equally effective, general cancer treatment. However, the path towards the development of such treatments remains elusive due to multiple technical challenges. Importantly, due to the complexity of bacteriomes, one needs to study the behavior of healthy and cancerous cells in the presence of complex bacterial communities and their combinatorial interactions, as opposed to the presence of a single bacterial species. This is because bacteria have species-/strain-specific effects [65–67], and are highly adaptive as their behavior is strongly influenced by the presence of other microbes within the population [68]. However, such investigations are obstructed by many challenges, including the difficulty of mimicking the natural host environment and the lack of high-throughput methods to study cancer cell-bacteriome interactions.

Here, we review the current state of the field with a focus on work that has been done to understand cancer progression in the presence of complex and naturally occurring human bacteriomes. We discuss currently available experimental approaches, challenges, and limitations to using these approaches, and possible routes to advance the study of cancer-bacteriome interactions. We further discuss the need for theoretical and mathematical frameworks to tackle the complexity of the human bacteriome. This problem needs strong interdisciplinary collaboration between microbiologists, geneticists, chemists, physicists, mathematicians, and data scientists. This paper is intended to highlight the need for developing new methods to study the interaction between cancer cells and the bacteriome and to present this problem in simple general terms to audiences across all scientific disciplines.

## Routes of bacteria-induced tumorigenesis

Several mechanisms have been reported in the literature through which bacteria can influence cancer development, including DNA damage, metabolic stimulation, inflammation, apoptosis regulation, or suppression of tumor suppressors [33, 34, 37, 69, 70]. In some cases, multiple mechanisms are responsible for cancer development [71]. Here, we discuss some of the pathways by which certain well-established “oncobacteria” induce cancer development. For example, *H. pylori* infection is believed to cause tumorigenesis by a complex cascade of interactions. These start by inducing macrophage and neutrophil accumulation in host cells, leading to increased production of reactive oxygen species (ROS), which in turn causes DNA damage and subsequently tumorigenesis [72, 73]. Additionally, *H. pylori* infection has been shown to increase the recruitment of cytokines, which locally induce chronic inflammation and the activation of tumor-promoting pathways such as the STAT3 pathway [70, 73, 74]. The STAT3 pathway affects many downstream processes such as proliferation and apoptosis inhibition [75, 76].

Another example includes certain *E. coli* strains that express polyketide synthases (PKs) [77], and produce a small molecule called colibactin, which is unstable and causes DNA damage via cross-linking [78]. Such DNA damage leads to mutations upon DNA repair, and the high rate of mutation of host cells in *E. coli*-rich environments leads to tumorigenesis via induced genetic instability [66]. *F. nucleatum* is another example that aids in secreting cytokines such as IL-1β, IL-6, and TNF-α, which lead to chronic inflammation that fosters cancer progression [79, 80]. *F. nucleatum* has also been shown to dysregulate the Wnt/ β-catenin signaling pathway, which leads to abnormal increase in cell growth and inhibition of apoptosis [81]. Lastly, *F. nucleatum* stimulates DNA damage in many ways, including halting DNA repair processes [82], thereby increasing genetic instability and cancer initiation. *Bacteroides fragilis* is a species where some strains express a toxin known as Bacteroides fragilis toxin (BFT) that binds to E-cadherin and promotes the Wnt/ β-catenin signaling pathway [82]. It has been shown that BFT stimulates MYC signaling [83], which is an oncogene that promotes cell proliferation [84]. In this fashion, *B. fragilis* also induces cancer initiation and progression by upregulating cellular proliferation and downregulating apoptosis.

In sum, there are multiple mechanisms by which a bacterium can increase the risk of cancer initiation. However, the vast majority of human-inhabited bacterial species have not been mechanistically investigated in relation to cancer. This is partly because there are no current high-throughput platforms that can identify bacterial species that influence cancer cells in the first place. Furthermore, bacteria are known to be highly adaptive, and interbacterial networks can further complicate the mechanisms of cancer influence. It is therefore important to first appreciate the complexity of the bacteriome in connection with promoting or suppressing cancer and direct research towards developing high-throughput methods that can screen bacteria for influence on cancer. Doing so will enable us first to extract common mechanisms of bacteria-induced cancer (DNA damage, chronic inflammation, apoptosis dysregulation, etc.) and then to devise strategies to block these downstream mechanisms of cancer promotion.

## The vastness of the human bacteriome and the need for cancer-bacteriome studies

**Fig. 1:**
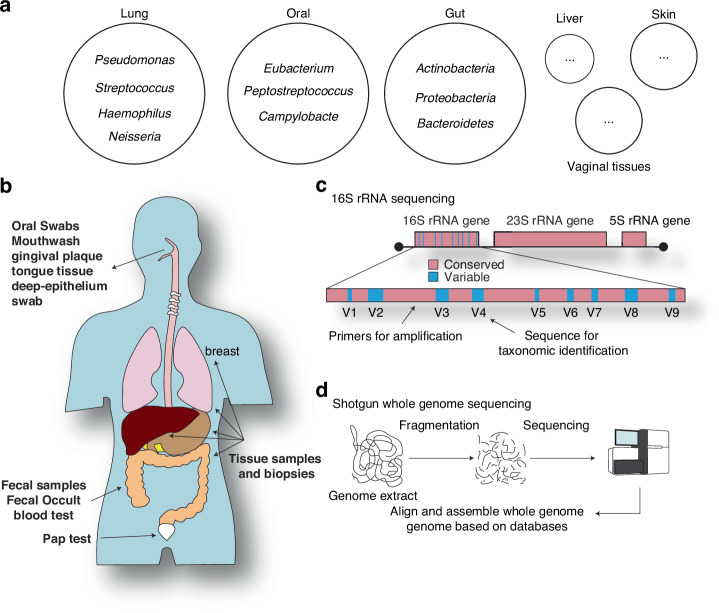
Bacteriome variability and available methods of analysis. **a** The variability of the human bacteriome based on the organ. **b** Sample acquisition methods at different organs for bacteriome analysis of different cancers. **c** A scheme showing the 16S rRNA gene and its conserved and variable regions. **d** Diagram showing the basic steps of shotgun whole-genome sequencing of microbial samples for microbial diversity and functional analysis.

Not only does the total number of bacterial cells in humans equal that of human cells [29], but there are thousands of bacterial species in the human bacteriome. Importantly, the bacteriome composition is associated with the overall health of an individual [2]. The local bacteriome composition depends on the tissue type and is also not homogeneous within the human population [27]. Distinct bacterial compositions are found in the lung [85–87], oral cavity [88], gut [89–91], and other organs [92–95] (Fig. 1a). These organs and other microenvironments represent complex ecological niches that influence bacterial adaptation and their interplay with cancer.

A recent review by Tekle and Garrett highlighted that the relationship between microbes and cancer is shaped by the broader microbial community, as coexisting species can modify the host’s susceptibility to oncogenic bacteria [33]. The authors highlight a study that showed that the combination of *Faecalibaculum rodentium* and *Holdemanella biformis* has an anti-proliferative effect on tumors in mice, irrespective of the other microbiota [96]. This highlights several interesting possibilities. First, it confirms the possibility that some components of the microbiome possess tumor-suppressive capability irrespective of the residing microbial community. It also highlights ecological concepts such as cooperation and competition in dictating a cancer-promoting bacteriome. Accordingly, *one should not only pursue cancer-bacterium interactions but also look at the inter-bacterial interactions that can affect the susceptibility of the host to cancer development*. Consequently, there is a dire need for cancer-bacteriome studies that mechanistically probe the interaction of cancer cells with this complex cocktail of bacteria.

Given the diversity of the human bacteriome and its dependence on host organs, there is a multitude of methods that have been developed to acquire bacteriome samples from different organs. These methods provide a window into the organ-to-organ variation in the bacteriome. Importantly, this complicates comparisons between different studies, as the method of obtaining the bacteriome sample may influence the results [97]. This also highlights the need for mechanistic and co-culture studies as opposed to correlation in the context of cancer. *The abundance of certain bacterial species that are associated with a higher risk of cancer does not automatically entail a causal link that is general to other organs* [98]. This may be why we see different bacteria associated with different cancers and not a single set of bacterial species that always correlates with cancer.

## Current approaches to studying the human cell-bacteriome

There are several studies that have probed the relation between cancer and the bacteriome as a whole. The bulk of these studies focused on bacteriome changes associated with the occurrence of cancer. However, few of them probed cancer evolution as a function of an increased abundance of one bacterium in the whole bacteriome population. We will discuss the methods used in these studies and highlight their strengths and limitations. Most bacteriome studies have been done in the context of oral cancer or colorectal cancer. A typical study of this nature includes sample acquisition, sample processing, and sample analysis via various sequencing methods.

### Sample acquisition

Acquiring samples from cancer patients usually includes tissue extraction and/or body fluids and decoding the bacteriome profile (Fig. 1b). Controls are generally healthy individuals or tumor-adjacent healthy tissue. In the context of oral cancer, sample acquisition can vary, such as tumor and tumor-adjacent tissues [99], biopsies and deep-epithelium swabs [100], and oral swabs and washes [101–104]. For colorectal cancer (CRC), most studies use fecal samples to analyze the gut bacteriome of cancer patients [105–112] in addition to tissue samples [113]. Caveats for sample acquisition include the variability of results based on collection methods and based on the variance of the bacteriome itself within different locations [114, 115]. With that being said, tissue samples are superior and are needed in order to map complete relations between the bacteriome and cancer. This is because the tumor microenvironment has a significant role to play in cancer progression and proliferation [116]. The tumor microenvironment has also been shown to contain a unique microbiome and is therefore another important aspect of bacteriome-cancer interaction that needs to be studied [14]. We assert that conclusions drawn from bacteriome-cancer studies must be carefully assessed in relation to the sample type and how it may influence the generalizability of the obtained results.

### Sample analysis

Sample analysis of bacteriome composition can be generally classified into two main methods: 16S rRNA sequencing and metagenomic shotgun sequencing. The 16S rRNA genes have several regions that are highly conserved throughout the entire bacterial tree of life. Interspersed among these regions of conservation are variable, species-specific regions (Fig. 1c). The conserved regions are used for primer design to amplify the variable regions using polymerase chain reaction methods. The variable regions are then sequenced for species identifications [117–121]. Metagenomic shotgun sequencing relies on sequencing the genetic material of the entire microbial population (bacteria, archaea, fungi, viruses, phages, etc.) and even human cells present within the sample without amplification [122, 123] (Fig. 1d). This is done by fragmenting the genetic content of a sample and then sequencing it all. The sequenced fragments are then either assembled or aligned directly to genomic databases to identify the species and their abundance in the population.

While both methods are useful, they differ in value. Legacy sequencing methods, such as amplicon sequencing (i.e., 16S rRNA), are cost-effective and quick to identify the composition of a bacteriome. However, these approaches lack the necessary resolution, and they do not allow for the isolation and/or culturing of the bacterial species of interest [124]. Metagenomic shotgun sequencing is significantly more expensive due to the need for more sequencing reads. However, it provides a more accurate determination of the bacteriome composition, especially concerning low-abundance species and all other microbial communities [124]. Metagenomics is therefore superior in most cases and should be used if available. The cost factor between the two methods can be mitigated if one can analyze a large number of samples at once. In other words, if a high-throughput method is developed where many bacteriome-cancer samples are collected and pooled together before analysis, one can increase the value of metagenomic sequencing by analyzing multiple samples in one sequencing run. This, of course, would need significant method development as we will discuss in the following sections.

## Defining the question of cancer-bacteriome interactions

**Fig. 2:**
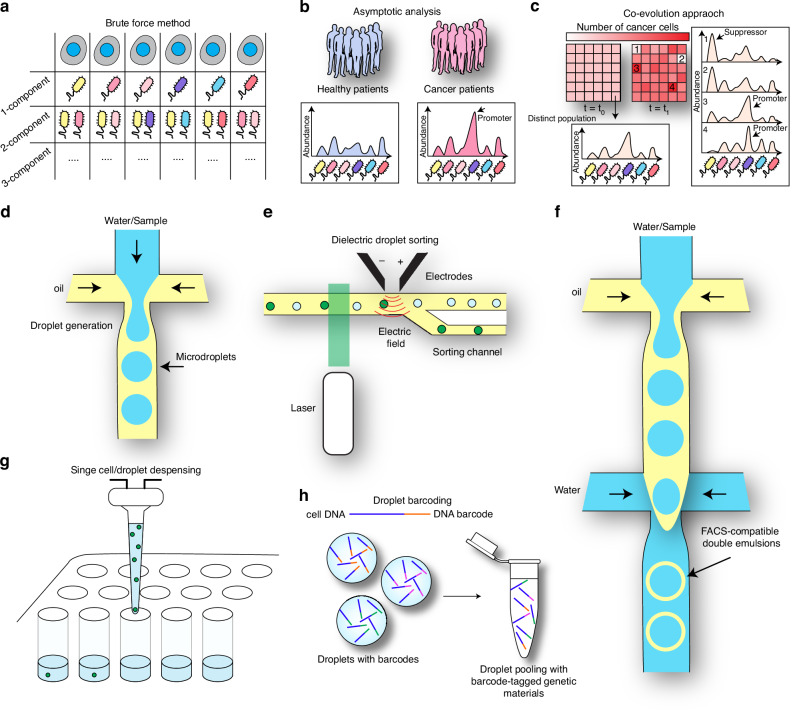
Approaches to solving the cancer-bacteriome problem. **a** Brute-force method where the permutations of bacterial species are tested individually. **b** Cancer promoters and suppressors can be identified by looking at the bacteriome of asymptotic cases of cancer patients and healthy patients. **c** Co-evolution approach where a large number of distinct bacteriomes are generated and the evolution of cancer cells within is tested. **d** A scheme of a flow-focusing microfluidic droplet generator to generate water-in-oil droplets. **e** An example of microfluidic droplet sorting using an electric field coming from electrical electrodes to deflect fluorescent droplets into the sorting channel based on fluorescence. **f** An example of how water/oil/water double emulsions can be generated. These double emulsions can be sorted using conventional FACS cell sorters. **g** A scheme illustrating the concept of single-cell/droplet dispensing. **h** Droplet barcodes allow for tracking the genetic material coming from each droplet via sequencing, even after the emulsion is broken and the droplet samples are pooled together.

The study of bacteriome-cancer cell interactions represents a complex, multivariate problem. Consider the promotion or suppression of cancer as a multivariable function $F({x}_{1},\,{x}_{2},\ldots ,\,{x}_{n})$ that depends on thousands of variables ${x}_{n}$ that include dependent and independent variables. There also exist interactions between those variables that can impact the overall outcome function. Each variable here represents a bacterial species within the bacteriome. The challenge is to map this function to predict the effect on cancer by looking at the bacteriome. To seek a solution for this problem, there are multiple approaches described in the literature. The simplest one is a brute-force method where we study the effect of each bacterial species on cancer cells (*i.e*., setting all the variables to zero except for one variable, Fig. 2a). Next, we study the effect of all possible two-component bacteriomes, and so on. While this approach would yield a complete mapping of the cancer function $F$, it is not feasible due to the resources it requires and the time it would take (hundreds of years).

Another approach is to look at the asymptotic behavior of the bacteriome at the extremes of the cancer function $F$. We may do this by looking at individuals who have cancer and analyzing their bacteriome (Fig. 2b). We then look at the individuals who do not have cancer and analyze their bacteriomes (Fig. 2b). After accumulating sufficient data, we can extract common features of the bacteriomes associated with cancer and those not associated with cancer (Fig. 2b). This is the current approach in the field and has resulted in identifying many bacterial species that do have cancer-promoting activity. However, there are limitations to this approach. First, it does not detect bacterial species that are conditionally cancer-promoting (i.e., exert a cancer-promoting effect when another species is present). Second, isolating species of interest that are responsible for the observed effect from a complex bacteriome community is not always possible. Furthermore, this method does not work well in detecting cancer-suppressive bacterial species because the definition of a healthy individual lacks accuracy. A person who does not have cancer is not necessarily healthy, and there is no reason to think that such a person would not develop cancer in the near future.

There is a third approach that can be used to understand the interaction between cancer cells and the bacteriome, which involves monitoring the co-evolution of cancer and bacteriomes in a co-culture setting. Briefly, one would track the growth and viability of cancer cells in a large number of distinct bacteriomes and select for bacteriomes that promote cancer cell proliferation as well as bacteriomes that suppress the growth of the cancer cell population (Fig. 2c). This approach is similar to brute force in principle, but it makes the entire process parallelized, which speeds up the discovery to a great extent. With that being said, in order for this approach to be feasible, it is required that we develop high-throughput methods for both the generation of bacteriome samples and screening them for cancer promotion or suppression. Without the high-throughput methods, this approach can only look at the relation between a cancer cell population and a few components, such as one or two bacterial species. We suggest that expanding research efforts into developing high-throughput screening methods for bacteria-cancer cell interactions is essential to develop a comprehensive understanding of the complex interaction hierarchy between cancer cells and the bacteriome. We speculate that such high-throughput methods will depend on the latest sequencing technologies, as well as significant advances in sample preparation, microscale culturing, and detection methods.

In sum, we can articulate three methods for probing cancer bacteriome interactions. The brute-force method looks at one species at a time and suffers from impracticality, high costs and labor, and limitations with regard to detecting complex and conditional bacterial networks. The second is large-scale asymptotic screening, where we take patients with clear cancer tendencies (i.e., developed cancers) and sequence their bacteriomes to extract common features of bacterial compositions. This is the most feasible approach currently, and it has been successful in detecting bacterial species with cancer-promoting activities [14, 23]. However, this approach is biased towards detecting only cancer-promoting bacteria and is oblivious to the mechanisms by which the said bacteriomes can increase cancer proliferation. A third approach that we suggest is high-throughput co-culturing of mammalian cells with distinct complex bacteriomes. This approach is currently not feasible due to the lack of experimental platforms that can generate a large number of bacteriomes and analyze their influence on cancer cells. However, this approach holds great promise since it can detect both cancer-promoting and cancer-suppressive species as well as conditional networks of bacteria that may promote or suppress cancer. We argue that this approach may be feasible if researchers are able to develop high-throughput co-culturing platforms. In the next section, we highlight microfluidic technologies that may prove useful for developing such miniaturized co-culturing platforms.

## High-throughput microdroplets for studying the bacteriome

High-throughput methods are necessary to effectively understand the complex interactions between the bacteriome and host cells. The difficulty in studying bacteriome-cancer interactions is that large volumes of data are needed for sample analysis and characterization and require high-throughput, multiplexed experimental techniques for sample and data generation and processing. Importantly, it is necessary to be able to generate a large number of compositionally distinct bacteriomes in order to study bacteriome composition features and their effect on disease. Of the emerging technologies, microfluidics has shown incredible promise for high-throughput scaling of currently established experimental technologies and can already be readily integrated with other experimental techniques. Microfluidics relies on the manufacturing of microfluidic chips [125]. The microfluidic channel design allows the generation of equally sized aqueous microdroplets in an organic liquid phase, such as oil (Fig. 2d). Millions of droplets can be generated, representing millions of independent/isolated samples, in a relatively short time (1–2 h). The analysis of these samples, however, is not straightforward due to their pico- and femto-liter volume. In order for microfluidics to be truly high-throughput, one must be able to analyze each droplet independently.

The first mode of analysis of microdroplets is droplet sorting [126], which can split the droplet population into two populations depending on a certain readout, such as fluorescence, light scattering, or other measurements. This allows the selection of a small percentage of the droplet population that exhibits a high trait (detected by fluorescence) [127] (Fig. 2e). Other microfluidic variations can be made to allow droplets to be sorted with conventional cell sorting flow cytometry (double emulsions, Fig. 2f) [128–130]. While droplet sorting can lead to bulk splitting of the sample population into smaller subpopulations, it does not achieve single droplet resolution, which significantly limits the applications of this technology. An advancement came with the advent of single-cell dispensing technology, which allows droplets/cells to be dispensed into 96-well or 384-well plates [131] (Fig. 2g). This improves the throughput, yet the size of the well plate poses a limit on the number of droplets that can be analyzed individually. A breakthrough advancement was achieved with the invention of DNA barcoding [132–134], which allows tagging the genetic material in each droplet with a short DNA sequence that is unique to the droplet (Fig. 2h). This DNA barcode allows for the identification of genetic material originating from each droplet individually via sequencing experiments without the need for unique sorting instruments, which significantly improves the throughput.

These developments represent a paradigm shift in high-throughput studies and have been used with immeasurable success in the study of the human microbiome. They can be used to detect and quantify bacteriome populations, study bacteria-host interactions via transcriptomics and RNA sequencing, and identify strains and genetic variants associated with cancer phenotypes. Below, we provide examples of the combined use of microfluidics with genomics methods that have been successfully applied to high-throughput microbiome characterization studies that are relevant to the study of bacteriome-cancer interactions.

**Fig. 3:**
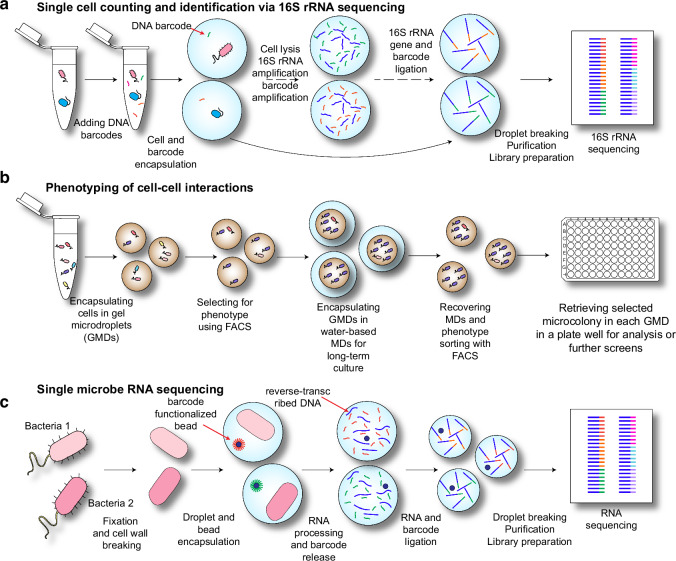
Microfluidics-based high-throughput methods for studying host cell and bacteriome interactions. **a** Single-cell counting and identification via BARBiq-seq [133]. **b** cell-cell interaction phenotyping by HiSCI [137]. **c** smRandom-seq workflow for single-microbe RNA sequencing [139].

Microfluidic high-throughput methods can be used for species detection. Zhang et al. sequenced ~20000 single-microbe genomes with strain resolution from the human gut microbiome [135]. They revealed 100 bacterial species, along with their sub-strain identification, and detected horizontal gene transfers and host-phage interactions from a single donor’s microbiome sample. In terms of microbial composition and counting, Jin et al. developed a method based on droplet microfluidics to identify and count single bacterial cells within the microbiome using a DNA-barcoding method called BarBIQ [136] (Fig. 3a). This method overcomes the limitations associated with quantifying bacterial species and features enormous throughput, in the range of 500,000 cells, as the authors reported. Ohan et al. used gel microdroplets (GMDs) as picoliter microenvironments to perform high-throughput screening of cell-to-bacteria interactions (HiSCI) using fluorescence sorting methods [137] (Fig. 3b). Other droplet-based techniques have also been deployed to enrich rare and slow-growing species in natural microbiomes via encapsulation, culturing, and sorting of micro-organisms [138].

Microfluidic high-throughput methods can also be used to perform RNA sequencing and transcriptomics to observe bacterial interactions and genetic profiles. There are multiple studies that successfully used high-throughput RNA sequencing to study bacterial transcriptomics with single-cell resolution. Xu et al. proposed a droplet-based high-throughput single microbe RNA sequencing method called smRandom-seq [139] (Fig. 3c). It is also possible to carry out complete biochemical reactions within microfluidic droplets. The same group has shown the possibility of doing single-cell transcriptomics using droplet technology by storing the PCR primers and cell barcodes into a hydrogel particle and mixing them with the cell suspension [133]. In such a case, the sequencing data could be split based on the barcodes, and hence droplet-specific transcriptomics are obtained. These and other examples illustrate the great potential for using microdroplet techniques to probe host-bacteria interactions. We believe that now is a great opportunity to harness the immense progress in droplet microfluidics and microbiology to address the problem of sampling the complexity of the microbiome, and couple this technique with high-throughput ‘omics data generation and analysis. These directions could advance the field forward by enabling high-throughput co-culture of cancer cells and distinct bacteriomes using droplet microfluidics as a platform.

## Analytical tools to analyze bacteriome-cancer interaction data

The tremendous volume and high-dimensional nature of the data obtained from high-throughput bacteriome-cancer interaction experiments have led to the development of a myriad of analysis techniques ranging from statistical ecological analysis to dynamical modeling. Empirical techniques like statistical analysis and some machine-learning algorithms attempt to sift through data to reveal underlying relationships. In contrast, “physics-based” or “mechanistic” modeling encodes a biological hypothesis and asks how well the data fit that hypothesis. This seeming dichotomy of “top-down” vs. “bottom-up” is actually a spectrum of strategies, with information often able to flow between modeling paradigms, such as when statistical analysis suggests relationships that can be encoded in dynamical models that then provide predictions to be verified by statistics. As with all computational biology, the right tool for answering any question about the bacteriome-cancer interaction will be highly dependent on the question being asked. In this section, we highlight a sampling of analysis tools that may prove useful for discovering patterns or revealing the interactions between the bacteriome and cancer.

### Dimensionality reduction

Dimensionality reduction techniques, such as principal component analysis (PCA), identify dependencies in a data set to remove noise and enable further analysis. Reducing the dimensionality of the data allows researchers to visualize data and is a key step to efficient downstream analysis [140]. Dimensionality reduction alone cannot reveal causal relationships within data, but it can suggest relationships between data features. For example, PCA has been used to identify biomarkers that best describe disease conditions, as well as distinguish whether a highly complex microbial diversity is closer to those of healthy patients or cancer patients [141]. Several other dimensionality reduction techniques, which are reviewed elsewhere [140], include both linear and nonlinear methods for projecting the data onto lower-dimensional spaces or manifolds in order to reduce noise and enable analysis.

### Statistical modeling and network inference

Statistical models can be used to draw conclusions and generate new hypotheses from data by comparing the data to the expected outcome from some random process. For example, multifactor analysis of variance (ANOVA) is used to determine the extent to which a predictor variable is able to affect the outcome variable [142, 143] by comparing the data to the expectation and variance of a general linear predictor model. It is mostly used when a single outcome variable depends on many factors. Multi-factor ANOVA computes not only the one-to-one relationship between each predictor factor and the outcome variable but also computes the interactions between the factors themselves, making it a potentially useful tool for analyzing large sets of data coming from high-throughput bacteriome-cancer cell interaction studies. Another class of statistical methods compares the data to a model that asserts that the bacteriome consists of several communities. Non-negative matrix factorization [144] or parameter fitting to more complex probabilistic models [145] can reveal the composition of these communities and their contribution to each sample of the data. These approaches can be especially powerful in identifying communities, rather than individual microbes, that may be associated with different cancer outcomes.

### Machine learning and artificial intelligence

The use of AI in microbiome research is vast and has recently been reviewed elsewhere [146–149]. AI methods have been used in many aspects of microbiome research, including taxonomic profiling [150], genome assembly and classification from sequencing data [151, 152], antibiotic resistance prediction [153, 154], and more. AI has also been used to identify microbial markers associated with cancers such as CRC [155–157]. These studies rely on existing data sets from cancer patients to construct models that can predict cancer based on the microbiome composition obtained from tissues or other sample sources. Another relevant application of AI includes predicting protein-protein interactions between bacteria and host cells [158, 159] and predicting microbe-disease associations [160, 161]. In fact, even large language models such as GPT-3 and BERT have been used to survey the literature for microbe-disease associations [162].

## Conclusion

Cancer has put a huge toll on human health and our economy [163]. Although there has been tremendous progress in our understanding of the genetic and epigenetic factors that govern cancer initiation and progression [164, 165], the rise in the number of cancer patients and the mortality rate remains high. The emerging link between cancer and the microbiome has recently allowed us to utilize and engineer microbes to detect and even fight cancer [13, 17, 18, 166]. Yet, our understanding of the interplay between cancer and the microbiome in the complex ecological system that is the human body is still limited. Here, we highlight the challenge of investigating cancer-bacteriome interactions and emphasize the importance of studying the bacteriome as a whole entity and probing its interactions with cancer cells. While some progress has been made in genetic engineering of individual bacterial strains to fight cancer, we propose that engineering the bacteriome as a whole, i.e., manipulating the population of natural species, may offer a more robust strategy to combat cancer. Apart from highlighting the challenges, we also review some of the approaches taken to better understand the impact of the bacteriome on cancer, speculating on the most appropriate and effective approaches, such as high-throughput co-culturing platforms. Consequently, we argue that high-throughput methods are essential to effectively probe and map cancer-bacteriome interactions and highlight some of the promising methods that can be used to study cancer-bacteriome interactions. To be able to effectively understand and then harness the interactions of our bacteriome with cancer, we also need to develop effective experimental and analytical tools that are capable of handling large volumes of complex, multidimensional data. To this end, we also review some of the multivariable statistical methods as well as the emerging use of AI in microbiology. Overall, there is significant potential for progress that could be made towards microbe-based cancer therapy by studying and understanding the relation between cancer and complex multi-species bacterial populations that constitute the bacteriome.

## Data Availability

No datasets were generated or analyzed during the current study.
